# Clinicians’ perspectives on supporting individuals with severe anorexia nervosa in specialist eating disorder intensive treatment settings during the COVID-19 pandemic

**DOI:** 10.1186/s40337-022-00555-4

**Published:** 2022-02-24

**Authors:** Hannah Webb, Bethan Dalton, Madeleine Irish, Daniela Mercado, Catherine McCombie, Gemma Peachey, Jon Arcelus, Katie Au, Hubertus Himmerich, A. Louise Johnston, Stanimira Lazarova, Tayeem Pathan, Paul Robinson, Janet Treasure, Ulrike Schmidt, Vanessa Lawrence

**Affiliations:** 1 PO59 Section of Eating Disorders, Department of Psychological Medicine, Institute of Psychiatry, Psychology and Neuroscience, King’s College London, London, SE5 8AF UK; 2 Department of Health Services and Population Research, Institute of Psychiatry, Psychology and Neuroscience, King’s College London, London, UK; 3 South London and Maudsley NHS Foundation Trust, Maudsley Hospital, Denmark Hill, London, SE5 8AZ UK; 4 Institute of Mental Health, University of Nottingham, Jubilee Campus, Triumph Road, Nottingham, NG7 2TU UK; 5 NHS Grampian, Aberdeen, UK; 6 South West London and St George’s Mental Health NHS Trust, London, UK; 7 Surrey and Boarder Partnership, NHS Foundation Trust, Surrey, UK; 8 Division of Medicine, University College London, 5 University Street, London, WC1E 6JF UK

**Keywords:** Anorexia nervosa, Clinicians, COVID-19, Eating disorders, Intensive treatment, Qualitative research, Telemedicine, Day patient, Inpatient

## Abstract

**Background:**

The COVID-19 pandemic has significantly affected intensive treatment settings (i.e., inpatient [IP] and day patient [DP]) in specialist eating disorder services. However, the impact on clinicians working in these services is largely unknown. We therefore explored the perspectives of those supporting individuals with severe anorexia nervosa (AN) in intensive treatment settings during the pandemic.

**Methods:**

Between May 2020 and June 2021, we interviewed clinicians (*n* = 21) who delivered IP and/or DP treatment to patients with severe AN in four specialist eating disorder services in the United Kingdom. Data relating to experiences during COVID-19 were analysed using reflexive thematic analysis.

**Results:**

We identified six themes: Disruptions to Routine Treatment; Introduction of Virtual Treatment; Separation from Treatment, Others and the World; Impact on Recovery; Impact on Staff; and Pressure on Referral Pathways. COVID-19 posed significant challenges to IP and DP services: forcing closures, operating with restrictions and virtual treatment, and impacting delivery of essential treatment components, referral pathways, clinician wellbeing, risk management, and patient isolation and recovery trajectories. Opportunities arose, in particular in DP services offering virtual support.

**Conclusions:**

COVID-19 challenged the continuation of multidisciplinary treatment. The findings underline the necessity for medical, psychological, practical, and nutritional support, as well as carer involvement and fostering social connections to remain at the forefront of intensive treatment for severe AN. They also emphasise the uncertainty surrounding which intensive treatment may be best suited to which patient when, particularly within the context of virtual DP support.

**Supplementary Information:**

The online version contains supplementary material available at 10.1186/s40337-022-00555-4.

## Plain English summary

The COVID-19 pandemic has significantly affected eating disorder inpatient and day patient treatment. However, the impact of the pandemic on clinicians working in these settings is largely unknown. We interviewed twenty-one clinicians working in specialist inpatient and day patient eating disorder services to explore their views on supporting people with severe anorexia nervosa during the pandemic. We analysed the transcripts using thematic analysis. We identified that COVID-19 posed significant challenges for intensive treatment settings, forcing the closure or merging of eating disorder services, the delivery of treatment under restrictions, and the introduction of virtual treatment. These changes challenged the delivery of multidisciplinary treatment for people with severe anorexia nervosa and impacted referral pathways, clinicians’ wellbeing, risk management, and patients’ isolation and recovery trajectories. We also identified some opportunities as a result of the pandemic, in particular in day patient services offering virtual support. These opportunities included more accessible treatment for patients and their families, more individualised treatment, and the chance for treatment innovation and creativity.

**Supplementary Information:**

The online version contains supplementary material available at 10.1186/s40337-022-00555-4.

## Introduction

Since the start of the COVID-19 pandemic, there has been an increased demand for eating disorder (ED) care and support [1, 2]. Measures to contain the spread of the virus (e.g., lockdowns, social distancing measures, and other restrictions), have significantly impacted the functioning of specialist ED services [3]. For example, reduced face-to-face contact has been necessary to protect patients and clinicians and, where possible, many services have adapted to provide healthcare online [4]. Nonetheless, the impact of such abrupt changes on specialist ED services is largely unknown. Questions around the impact of COVID-19 are particularly relevant for intensive treatment settings (e.g., day patient [DP] and inpatient [IP]) for whom adapting intensive delivery within COVID-19 restrictions poses significant challenges.

Limited research has considered clinicians’ experiences of the pandemic, with most focusing on the patient and/or carer perspective [5–7]. To date, clinicians’ experiences of delivering ED treatment during the pandemic have been explored in a service evaluation of a young person’s ED service [3] and in an online survey of clinicians working in ED services in the initial stages (March–May 2020) of the pandemic [8]. These studies suggest COVID-19 has significantly impacted ED service delivery. Clinicians raised concerns over their own lack of support and uncertainty, therapeutic inefficiency and compromised therapeutic alliance, managing high risk patients remotely, and increases in patients’ dysfunctional behaviours and feelings of social isolation. Conversely, clinicians also identified some opportunities, such as increased frequencies of communication with patients/families and greater ability to foster patients’ autonomy when working remotely. Whilst pertinent, no research has yet explored the impact of COVID-19 on clinicians supporting adults with severe AN specifically in intensive treatment settings. Therefore, using semi-structured interviews, we aimed to explore clinicians’ perspectives and experiences of supporting individuals with severe AN in intensive treatment settings during the COVID-19 pandemic.

## Methods

Ethical approval was granted by the Wales Research Ethics Committee 5 (Reference: 20/WA/0072). Data collection for this study was performed as part of the process evaluation of the DAISIES trial (see 9, for protocol); an ongoing two-arm multi-centre open-label parallel group non-inferiority randomised controlled trial evaluating the clinical and cost-effectiveness of IP treatment as usual and a stepped-care DP treatment approach (DP treatment with the option of an initial IP treatment for medical stabilisation) for adults with severe AN.

### Participants

We interviewed twenty-one clinicians who, during the pandemic, delivered intensive treatments (IP and/or DP) to individuals with severe AN across four specialist National Health Service (NHS) ED Services in the United Kingdom (UK) (*n* = 17 from London-based services; *n* = 2 from a South-East England based service; *n* = 2 from a Scottish based service). Clinicians represented a purposive sample that sought diversity of professional background, years of experience in EDs, and ED setting (although participation was informed by clinician interest and availability), from selected specialist ED Services involved in the DAISIES trial [9]. Recruitment continued until the sample was deemed to hold satisfactory information power, in line with our broad aim and interest in exploring a range of perspectives [10]. See Table 1 for demographic details.

**Table 1:** Setting, role and years of experience in eating disorders for each participant

Participant and setting (OP, DP and/or IP)	Role	Years of experience in EDs
P1-OP	Consultant Psychiatrist	10 +
P2-IP	Consultant Psychiatrist	5–10
P3-IP	Consultant Psychiatrist	10 +
P4-DP	Occupational Therapist	5–10
P5-OP/DP	Consultant Psychiatrist	10 +
P6-OP	Clinical Service Manager	10 +
P7-DP	Nurse Specialist	0–5
P8-IP	Consultant Psychiatrist	0–5
P9-DP/IP	Nurse Therapist	10 +
P10-OP/DP	Dietician	10 +
P11-OP/DP	Nurse Specialist	0–5
P12-IP	Counselling Psychologist	Not reported
P13-DP	Mental Health Nurse	0–5
P14-DP	Assistant Psychologist	0–5
P15-IP	Counselling Psychologist	10 +
P16-DP	Assistant Psychologist	0–5
P17-DP	Mental Health Nurse	0–5
P18-OP/DP	Occupational Therapist	0–5
P19-DP	Occupational Therapist	0–5
P20-OP/DP	Consultant Psychiatrist	10 +
P21-DP	Day Unit Manager	10 +

*OP* outpatient, *DP* day patient, *IP* inpatient, *ED* eating disorder

### Data collection

Audio-recorded semi-structured interviews were carried out by researchers (BD, DM, MI, and GP) via Microsoft Teams, at a time convenient for each participant, between May 2020 and June 2021. Interviews lasted approximately 50 minutes, were transcribed verbatim and identifiable information was removed.

The topic guide (see Additional file 1) was designed by authors MI, US, VL and BD. The interview had broader aims, outside of the current analysis, to explore clinicians' views and experiences of managing individuals with severe AN in intensive treatment settings. However, owing to the UK Government-imposed COVID-19 restrictions (which commenced on March 23^rd^, 2020), questions were added to explore clinicians’ perspectives of the immediate and future impact of the pandemic on how they support those with severe AN in their services. Questions were asked in an open manner with the researcher providing encouragement, probing and seeking clarification of answers to explore participants’ concerns. For the present study, only data relating to the COVID-19 pandemic are reported (see 11, for analysis of the broader data).

### Data analysis

We analysed the data using reflective thematic analysis (supported by NVivo 12). Acknowledging the significance of the participants’ and researchers’ understanding of the phenomenon under exploration, we took an inductive, interpretivist approach and followed the six phases outlined by Braun and Clarke [12–14]. In brief, transcripts were read and reread to ensure data familiarisation. Two researchers (DM and VL) double-coded three transcripts and through collaborative discussions, sorted initial codes into themes. From this, an initial coding framework was generated, and discrepancies were considered until a consensus was reached. A third researcher (HW) then reviewed this and, through ongoing discussions (with VL) and a continuous process of refining and defining themes, developed the final coding framework. The analysis was then summarised into a written reported by the first author (HW) and circulated and approved by all authors. HW (mental health researcher, practitioner and former service user) and VL (social scientist) drew upon past experiences of conducting qualitative research (in EDs and mental health more broadly). HW also reflected throughout on how her knowledge and experience of ED treatment might influence her approach to the data. For each theme, a summary of participants’ perspectives is provided with supporting quotations, anonymised using participant numbers suffixed with participants’ current work setting (i.e., OP, DP and/or IP).

## Results

**Fig. 1:**
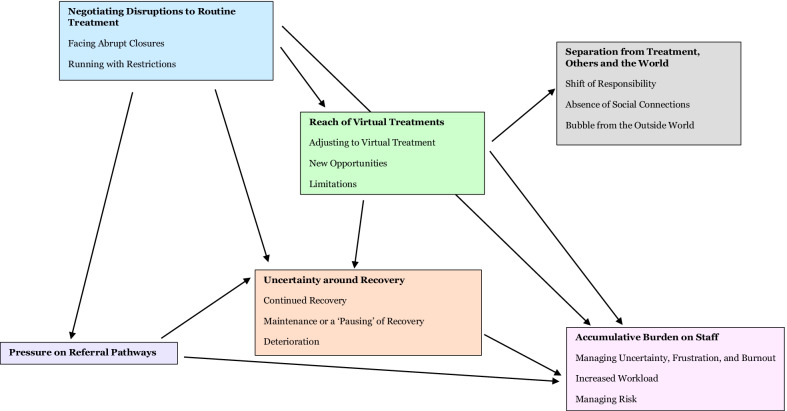
Thematic map

Six themes were generated from the data (see Fig. 1 for a thematic map): Negotiating Disruptions to Routine Treatment; Reach of Virtual Treatments; Separation from Treatment, Others and the World; Uncertainty around Recovery; Accumulative Burden on Staff; and Pressure on Referral Pathways.

### Theme 1: negotiating disruptions to routine treatment

#### Facing abrupt closures

Prevalent among clinicians’ accounts were descriptions of sudden changes to usual treatment.

A lot of knee jerk reactions… we didn't know how long it was going to last. (P16-OP/DP)

Clinicians in both IP and DP settings described closing services, discharging patients, and declining new referrals. Many voiced discomforts over discharging patients when “they were not safe and not stable” (P3-IP/DP). One clinician described how their IP ward was merged with another service with “a very different culture and quite crowded circumstances” (P1-OP) and several had concerns about patients who had been transferred.

That was very traumatic for them…. it was traumatic for the team as well…. (P12-IP)

#### Running with restrictions

Most frequently mentioned by IP clinicians were concerns and frustrations over leave and visitation restrictions: patients were no longer able to go on walks, day/overnight leave, or have visitors. One clinician explained how reduced time outside provoked increases in ED behaviours (e.g., pacing, standing, self-harm). Several others mentioned worries over reduced contact between patients and carers. Extra thought was given to out-of-area patients, whose parents “aren't going to be as likely to come down if … they're (only) allowed in for an hour” (P9-DP/IP). IP clinicians described difficulties in maintaining patient motivation without incentives and in supporting phased transitions.

IP clinicians also described having to change ward and/or dining room layouts to adhere to social distancing/self-isolation requirements, adjust to wearing personal protective equipment (PPE), and pause, limit, or make virtual, various psychology and practical food-related skills groups. Most IP clinicians described difficulties with meal support: they were unable to eat with patients, had to wear masks and sit at a distance. This limited their ability to provide support or model normal eating behaviours. Two clinicians raised concerns over reduced psychological support, describing how “psychology can be pushed out, as if that's a bit of a luxury…” (P15-IP). Many hoped restrictions were temporary.

I very much hope that we're going to be able to eat with patients again, and they're going to have all those practical practise opportunities… because we know in our heart of hearts, they're not fully prepared at discharge... (P15-IP)

DP clinicians also described challenges due to the restrictions. All DP services transitioned to virtual support or a hybrid model of virtual and face-to-face support. As a result, some services reduced capacity and treatment days and/or hours. Like IP clinicians, DP clinicians were concerned about the lack of/changes to meal support and rest periods when working virtually. Clinicians voiced how crucial these were and suggested patients and clinicians struggled with their absence.

Being in person… they've constantly got a staff member there… That's something that quite a few people are really missing… they have identified that they really need that. (P13-DP)

A few services introduced virtual meal and post-meal support later, with one service labelling these “social eating groups” (P19-DP). Clinicians described challenges around attendance and not being able to see or control what patients were eating. A minority valued this addition, though recognised that it relied on “highly motivated people” (P1-OP).

Those anchor points were disappearing and the eating disorder's telling them … they didn't really need to have it, or they were eating alone and struggling. So, this gives a permission to eat and a focus and… gives a social aspect. (P4-DP)

Also frequently mentioned by DP clinicians were frustrations over being unable to do activities with patients, such as food exposure tasks, grocery shopping and cooking groups. Concerns arose over patients’ learnings being “way slower online” (P18-OP/DP) without hands-on in-person support.

One of the biggest things is the practical side of it, because, obviously, you can't do that virtually. So, there's been a lot of losses… the physical elements, the practical elements. (P17-DP)

For DP clinicians providing face-to-face treatment, ensuring social distancing within restricted spaces and equity of access (i.e., which patients should attend on which days/times) as “patients can be quite competitive…” (P14-DP) was difficult. Several expressed discomfort over in-person meal support limitations: they were unable to sit with patients and introduced individual tables and perspex screens. DP clinicians emphasised the importance of continued in-person medical monitoring (where possible). For example, one service saw patients in-person solely for physical observations and another sent taxis so that patients could visit at given timeslots. Again, many articulated a desire for these restrictions to be temporary.

### Theme 2: reach of virtual treatments

#### Adjusting to virtual treatment

All DP clinicians described introducing virtual support, and several outlined revising and changing their approach, depending on current guidelines/restrictions. Clinicians spoke of working “to adapt it, to improve it” (P13-DP) and “making a lot of changes” (P14-DP). Some services initially began with a “limited (virtual) day programme” (P1-OP) (e.g., only telephone calls), but as the pandemic progressed, the majority switched to more-or-less full-time virtual treatment or a blended treatment approach. The virtual offer comprised of individual and group psychological support, one-to-one sessions (e.g., with consultants, keyworkers, dieticians, or occupational therapists), creative, peer-to-peer and meal support groups, ward rounds and family work.

What we’ve done is basically try to reinvent our service into a virtual service, without having to think about it too hard and just do it… (P4-DP)

One DP clinician recognised that programme hours were reduced (compared to pre-COVID-19), but felt that, positively, this gave individuals time to focus on life outside of their ED.

It’s not as full on as when we were in person… so it does give them that flexibility to, it’s not constantly about treating an eating disorder, they can do other things in their spare time. (P13-DP)

Several IP clinicians described introducing some aspects of virtual support during the pandemic. These were new treatment elements and comprised of virtual meals with carers, individual psychological support (in preparation for virtual DP/OP treatment) and group support (as social distancing prevented accommodating all patients in one group room).

We’ve tried to do things like setting up video snacks, video meals, family sessions by video (P2-IP/DP)

Generally, clinicians felt patients and families adjusted well to virtual support especially “the younger ones who have no problems using computers” (P20-OP/DP), although some had found this transition challenging and “have been quite critical” (P2-IP/DP). Patients’ preferences and feedback had been at the centre of clinicians’ adaptations, through individual consultations and community groups.

#### New opportunities

DP clinicians felt virtual working provided opportunities for improved treatment accessibility for patients, carers, and the wider clinical team (e.g., to attend reviews) “who couldn’t necessarily commit to being face-to-face” (P13-DP). Several suggested that virtual support for patients reduced the potential for non-attendance, describing how previously patients’ ED behaviours or lengthy commutes had presented barriers to treatment engagement. In particular, many noted virtual treatment’s potential to support out-of-area patients—“the physical distance, it doesn’t matter anymore” (P4-DP)—and several suggested that those with social anxiety, autism, or physical health vulnerabilities may prefer virtual support.

Many DP clinicians similarly described how virtual working improved access for carers, enabling them to attend carer workshops, family sessions and reviews more easily. A few IP clinicians valued the “new way of (virtual) working” (P3-IP/DP) with carers and suggested they will continue to use the “lovely new tech set-up” (P15-IP). Notably, several services had increased carer support and wished for this to continue, wanting “to make it very much part of the treatment… not just an afterthought” (P6-OP/DP).

We involve the families much more now… because most of them are also at home… there is much more support available. (P5-OP/DP)

A few DP clinicians felt virtual treatment facilitated a “more individualised” (P5-OP/DP) approach, with a relaxation of certain treatment boundaries to accommodate patients’ needs, an increase in one-to-one time (due to more virtual check-ins and/or therapy sessions), and a greater understanding of “what the patients really need” (P13-DP). Virtual treatment was better embedded within patients’ own environments—clinicians could make treatment more relevant to individuals’ lives, and patients could face difficulties directly with support and translate skills/learnings more smoothly. One clinician felt that being away from the hospital environment was also valuable.

It takes away some of the competitiveness… I think that's probably quite beneficial to be away from that and more focused on personal recovery. (P14-DP)

One DP clinician described doing a home visit and voiced that they wished for this to be a future possibility, and another had introduced acceptance and commitment therapy sessions for those awaiting individual therapy and explained how “getting them ready, like psychologically, for treatment has just been really helpful” (P16-OP/DP). Others described new groups that had been created, with suggestions that they would like these to continue, including problem solving and troubleshooting groups, and a group that encouraged “thinking about their identity that is outside of their eating disorder” (P16-OP/DP). Evidence of peer-to-peer support within these groups was particularly welcomed. Several also described the positives of a new creative group that allowed patients to connect socially while doing something they found personally enjoyable.

It gives them permission to sit down and do a focused leisure activity that they would normally struggle with because of the idea they're not being productive or that they feel lazy. (P4-DP)

Underpinning narratives from clinicians in both settings was the desire for certain opportunities to continue after the pandemic, and two clinicians specifically mentioned the potential of a “blended model” (P1-OP, P4-DP).

#### Limitations

DP clinicians described difficulties monitoring patients’ physical health when working virtually. Many had to rely on patients’ self-reported weights, which is “something we [clinicians] would never normally do” (P7-DP) and provoked anxiety.

As described, meal support was difficult to transfer online. DP patients “weren’t particularly keen” (P21-DP) and attendance was poor. Similarly, whilst IP clinicians described supporting patients to have virtual meals with carers, several voiced “it’s not the same” (P9-DP/IP) and “a feeling that it’s not good enough” (P12-IP).

DP clinicians described difficulties in ensuring patients had private spaces to engage in virtual treatment, with some expressing that virtual support is not accessible to everyone, especially those in small/shared spaces or who “can’t use computers or mobile phones” (P20-OP/DP). In addition, a few raised concerns over poor Internet, their own “glitchy system” (P10-OP/DP) and patients who refused to put cameras on. Indeed, several voiced apprehensions over taking on new patients remotely.

That causes a lot of anxiety for our original patients… some even expressed resentment around people joining that they’d never even met and having to open up to them. (P7-DP)

Indeed, IP clinicians also described practical challenges around virtual working, with several explaining how it took their services considerable time to set up adequate technology resources. One also articulated the difficulty of suddenly having to transfer to virtual psychological support.

My whole training is based on sitting in a room… there's just a lot that you miss doing it virtually… for some patients they hate it… (P12-IP)

### Theme 3: separation from treatment, others, and the world

#### Shift of responsibility

Clinicians in both settings suggested the pandemic (and discharges/virtual support) had prompted a shift in responsibility. Clinicians described increased pressure on carers to support their loved ones at home due to early discharges from IP treatment or DPs being supported virtually. For many carers, this was a sudden adjustment, as they had to “take on a much bigger role… much sooner” (P17-DP), and provoked unease. DP clinicians also described how the pandemic increased patient’s responsibility for their recovery, for example, due to the lack of/reduction in meal support, more control over their free time, and self-reporting of weight. One clinician suggested this meant changes were more sustainable for some, but slower/fewer for others.

In terms of how they're spending their free time… the gaps between group times, you know, we don't know what's going on, we don't have eyes on them, which is a challenge. (P17-DP)

#### Absence of social connections

IP clinicians suggested IPs were isolated from family/friends due to restrictions around leave and visitations. This was described as “extremely distressing” (P8-IP) for patients and carers.

DP clinicians suggested DPs also struggled with social isolation, particularly those who lived alone or who struggled before COVID-19—the pandemic “intensified their difficulties” (P11-OP/DP) and limited opportunities to foster a social/vocational life. Concerns were raised over patients having to manage tricky carer/family dynamics and the limited patient-patient and clinician-patient opportunities to connect informally during virtual treatment.

It's not only the formal programme… patients bond with each other and support each other… it's not quite the same and they are missing it. (P5-OP/DP)

Conversely, some DP clinicians suggested certain patients were more comfortable, due to the security of being in their own environments and the ease at which they could avoid their fears/difficulties. A few voiced apprehensions over how patients will manage the transition back to pre-pandemic life.

#### Bubble from the outside world

Two IP clinicians suggested some IPs had become disconnected from the outside world and resistant to discussing the reality of the pandemic. Upon discharge, some patients “had no idea what the reality was” (P6-OP/DP) (e.g., empty streets, supermarket queues). Although patients had seen/heard the news and one team had introduced a current affairs group to “mitigate some of the institutionalisation” (P2-IP/DP), patients expressed wanting to avoid thinking about “how bad things are” (P2-IP/DP).

### Theme 4: uncertainty around recovery

Descriptions of the impact of the pandemic on patients’ recovery trajectories arose solely in DP clinicians’ accounts. However, one clinician, working across IPs and DPs, emphasised the uniqueness of each patient’s journey.

It’s been really interesting to see the ones who've done really well and coped really well and the ones who really haven't and I'm not sure that I would've predicted correctly which would've done well and which would've done badly. (P2-IP/DP)

#### Continued recovery

DP clinicians described how some patients had adjusted well to remote treatment, were engaged, and continued to gain weight. One clinician felt the pandemic had impacted patients positively, giving them time to “look inside… and just really have a think about their recovery” (P16-OP/DP).

#### Maintenance or a ‘pausing’ of recovery

Others, however, described how few patients had improved clinically during the pandemic.

People aren’t gaining weight as they would have, but certainly maintaining. (P9-DP/IP)

Several spoke of how DP services had changed their expectations (e.g., weight gain requirements) as they transitioned to virtual support. These clinicians recognised the impact that effectively ‘pausing’ treatment had had.

It also paused their progress, that they just stayed where they were. It had to do with our stance as well, that we were not expecting them to make much progress during lockdown, which was probably a mistake, because it reflects on the patient... (P5-OP/DP)

Indeed, one clinician felt “treatment has to gain momentum again” (P3-IP/DP) and another noted the challenge of supporting patients to make changes when working remotely.

#### Deterioration

Several clinicians described how some patients had deteriorated. A few suggested patients “initially seemed to cope alright… and then they deteriorated” (P1-OP), with several having to be stepped-up to IP treatment. Others described how they were receiving referrals for “more physically ill patients” (P20-OP/DP), including those being treated as OPs and those completely new to ED services.

### Theme 5: accumulative burden on staff

#### Managing uncertainty, frustration and burnout

Clinicians in both settings described challenges around managing ongoing uncertainty and frequent changes, both for themselves and their patients. For some, learning to sit with uncertainty had “been a bit of a bonding experience” (P12-IP), bringing the ward community together; for others, it had caused continued anxiety. One IP clinician spoke of frustrations at the ongoing restrictions (when the first lockdown had ended), their impact on treatment and concerns over staff burnout.

People have not had holidays for a long time… everybody is getting a bit tired. (P2-IP/DP)

Several described challenges around navigating changes when “there wasn’t really a protocol or a guidance” (P2-IP/DP), voicing the complexity of deciding whether the risk of discharging patients into the community/treating them virtually was greater than the risk of patients experiencing a COVID-19 outbreak on the ward/day unit.

#### Increased workload

Several DP clinicians suggested virtual working increased their workload—everything was “more elongated” (P18-OP/DP). For example, one described how what would previously have been ‘a quick chat’ was now a “more formal toing and froing” (P10-OP/DP) and another described more frequent (email) communication with patients. One clinician also noted the positives of being more available, particularly for the most unwell patients. Moreover, one DP clinician spoke of how they and another senior member of staff were now providing therapy for all patients (even those not receiving therapy previously).

#### Managing risk

Several DP clinicians suggested the pandemic had forced them to manage more risky DPs (predominately remotely), in part due to the speed in which patients were transferred out of IP services and patients in the community having nowhere to go.

It's a case of really picking carefully who is priority for those beds (P14-DP)

To manage these risker patients, one clinician described being transparent about their concerns, ensuring close monitoring, and clear communication of expectations. Many had triaged patients, only seeing the riskiest patients in person and asking others to be seen by General Practitioners (GPs) who “would sometimes refuse to see people” (P3-IP/DP) or utilise family support. One DP service had introduced text/email crisis support as they felt it inappropriate for “junior staff who are not registered clinicians to manage patients remotely” (P21-DP).

They're like BMI thirteen or something, which when you're not seeing people face-to-face is really hard and risky… even if they come to physical monitoring… they're at risk all the time… it was a lot of pressure for us. (P19-DP)

### Theme 6: pressure on referral pathways

Clinicians in both settings described increased referrals, closing to new/national patients, and lengthy waiting lists (due to staff shortages, ward closures/merges and capacity limitations).

We have accumulated a waiting list of… more than 600 patients, and we don't know how we can reduce this… and how we could see so many patients (P3-IP/DP)

Clinicians particularly expressed concerns about reductions in capacity, describing a national shortage of IP beds, and for one site, reliance on a medical ward.

It's literally one out, one in… we may have to go nationally to find the bed…. (P6-OP)

Clinicians across both settings also described concerns over reduced primary care and OP/DP support during the pandemic.

Outpatient services here are limited… there's probably a lot of people really struggling in the community but are, kind of, being not picked up or GPs aren't seeing people face-to-face (P9-DP/IP).

IP clinicians described how, for patients who had not been discharged in the first lockdown, admissions were generally longer. DP clinicians described a similar pattern, due to treatment being ‘paused’ or run at reduced intensity. Contrastingly, a few clinicians suggested patients were being stepped down from IP to DP treatment more quickly.

Hospital admissions are probably longer. Because they've got to be in a good place to go rather than practising it and seeing what happens. (P9-DP/IP)

## Discussion

We explored clinicians’ perspectives of supporting individuals with severe AN in specialist ED intensive treatment settings during the COVID-19 pandemic. COVID-19 posed formidable challenges, forcing closures, operating under restrictions, and the introduction of virtual treatment. DP services, in particular, had to adapt their offer due to face-to-face contact within these contexts being largely prohibited. Across both settings, these changes impacted referral pathways, clinicians’ wellbeing, management of risk, and patients’ isolation and recovery trajectories. DP clinicians, compared to IP clinicians, described more opportunities as a result of these changes, highlighting the potential of (partially) virtual support as more individualised and accessible. Importantly, the impact of COVID-19 occurred within resource-limited services where referrals and the demand for intensive support were already on the rise [15, 16]. Despite ongoing challenges and uncertainty, a sense of rallying and responsive adaption was prevalent.

A key focus of multidisciplinary support for severe AN is nutritional rehabilitation [17, 18]. Yet, the pandemic disrupted meal support across intensive settings. In IP services, clinicians were unable to model normal eating behaviours or provide, what they described as, satisfactory therapeutic support. In DP services, meal support was often the last treatment element to transition online. DP clinicians expressed concerns over their ability to monitor attendance, food intake, or post-meal rest periods, and create supportive virtual dining environments. In both settings, the resulting constrained environments likely impacted the efficacy of therapeutic meals and the therapeutic relationship [19, 20]. Whilst research into IPs’ perspectives on COVID-19 is lacking, DPs suggest virtual (compared to in-person) meal support is less helpful [21] and a DP service labelled it the “biggest challenge to the continuation of care” (22, p.3). Given the importance of re-feeding for individuals with severe AN, future research is required to ensure safe, therapeutically supportive, and sufficiently monitored meal support is possible, both virtually and within restricted intensive settings.

Another key component of intensive treatment is (food-related) practical skills groups and exposure tasks. These support individuals to improve and challenge maladaptive ED thoughts and behaviours and transfer skills to life outside of intensive treatment, and thus provide a foundation for sustained recovery [23]. The pandemic hampered DP and IP services’ abilities to carry out these activities and limited patients’ opportunities to develop and practice transferring skills. Some clinicians suggested DPs’ permanency in their home environments better facilitated the transfer of (albeit limited) skills, consistent with research suggesting DP, compared to IP, treatment may have greater applicability and allow for a better transfer of skills from treatment to one’s life [11, 24]. Despite success in transitioning aspects of intensive support to virtual delivery, patients may prefer in-person equivalents [21, 22]. This emphasises the importance of the practical and psychological elements of AN treatment in both intensive settings and furthers calls for research and development of virtual ED support [25].

Social difficulties are a risk and maintaining factor in AN [26, 27], making individuals with AN particularly vulnerable to COVID-19 restrictions and the associated isolation [27]. Across both settings, concerns arose over patients’ limited opportunities to foster social connections. In IP settings, patients were isolated from friends/family and (socially/physically) distanced from clinicians, factors that may intensify social isolation, reduce patient engagement and autonomy in recovery, and thereby exacerbate IP institutionalisation [11, 28]. This finding echoes research into adolescents’ and their carers experiences of IP treatment during the pandemic; COVID-19 restrictions meant contact between patients and families, and families and clinicians, was perceived as limited and more emotionally burdensome [29]. Due to the virtual DP offer, the pandemic limited opportunities for patient-patient and clinician-patient connections, in addition to wider restrictions in the community. Some DP services created novel online groups to foster connections. However, clinicians generally felt opportunities to build relationships were limited, particularly for new patients joining remote treatment. These findings are consistent with patient and carers’ perspectives: whilst virtual support is appreciated, relational disconnect appears prevalent for patients, carers, and clinicians [21, 30]. These findings highlight the importance of encouraging social connections during intensive treatment, as well as greater consideration of how to foster virtual social connections and positive therapeutic relationships [20, 26].

Due to earlier discharges and/or virtual support, clinicians in both settings recognised the pandemic had shifted responsibility for recovery more towards patients and already burdened carers [7]. This shift appeared most notable in DP settings and corroborates research into Italian clinicians’ perspectives—virtual support may afford greater opportunity to foster patients’ autonomous motivation and more collaborative treatment [8], which have been associated with a greater reduction in ED behaviours [31]. DP, compared to IP, treatment appears to better facilitate responsibility in recovery [11, 32], thus, virtual support may further increase this opportunity. Nonetheless, whilst this shift appeared helpful for some, it was unhelpful for others. In particular, previous findings suggest virtual support may enable greater opportunity for compensatory behaviours and information secrecy, thereby contributing to patient deterioration [33]. Thus, a thorough assessment of patients’ motivation and readiness to change may be helpful in identifying the appropriate type of intensive treatment [34], particularly when engaging in virtual support. Moreover, due to DPs increased dependence on carers [7, 33], ensuring sufficient carer support during this time is also especially important.

Throughout clinicians’ narratives was a lack of certainty regarding which patients would cope during the pandemic. Virtual (or partially virtual) DP treatment supported continued improvements for some, echoing research which suggests that whilst challenging, certain patients’ benefit from virtual intensive support [5, 22]. Nonetheless, clinicians suggested many patients struggled to gain, or lost, weight during the pandemic; these difficulties have been reported globally [6, 8]. Indeed, COVID-19 added considerations and pressures on decision-making and risk management in intensive settings. IP clinicians described how capacity limitations and staff shortages affected patient length of stay and discharge parameters. DP clinicians expressed concerns around managing risk remotely, noting an increase in higher risk referrals. In addition, clinicians in both settings raised concerns over reduced community support (i.e., GP/OP), delaying recognition and access to specialist support, and thereby contributing to longer duration of untreated ED and potentially poorer outcomes [35]. Whilst elements of virtual ED support may be possible and promising, given the tendency to deny illness severity, treatment ambivalence, and frequency of relapse and physical health concerns in AN [36], clinicians concerns are unsurprising. Indeed, previous studies have raised concerns over remote risk management and reliance on patients’ self-reporting their weight during the COVID-19 pandemic [29, 37]. Due to frequent medical complications and difficulties surrounding patients’ self-reporting weight, medical monitoring remains integral, and precedence should be given to in-person physical health monitoring where possible. Interestingly, some DP clinicians reported pausing treatment expectations during their initial COVID-19 response. Whilst this may be unhelpful for some patients (e.g., reducing motivation for change), this may be considered a harm-reduction approach in light of uncertainty and significant change [38]. Adjusting treatment boundaries may permit more accessible DP treatment (accommodating individuals’ unique needs and recovery trajectories) and highlights the importance of an individualised, patient-centred approach, whilst ensuring that in-person physical health monitoring remains a priority [39]. Combined, these findings underline the need to better understand which patient may benefit from which intensive treatment and when [9], to support patients to access timely, appropriate, and effective intensive ED support.

Whilst COVID-19 caused many challenges, several opportunities also arose. Clinicians in both settings suggested virtual treatment increased access. For patients, it reduced geographical (e.g., travel time/expenses, locality limits) and comorbidity (e.g., for individuals with autism or social anxiety) barriers. This increased accessibility, which is consistent with research into virtual OP/DP ED support (e.g., 3, 21, 22, 37). Virtual treatment also enabled easier access to and greater provision of carer involvement and support (in both settings), corroborating previous research into adolescents with AN and their carer’s perspectives [21, 29]. This is of particular importance given the significant carer burden and distress often experienced, and the value of carer support for individuals with AN [40]. Moreover, concurring with other ED clinicians’ perspectives [8], virtual treatment supported wider multidisciplinary team attendance (e.g., at patient reviews) and increased the frequency of one-to-one encounters with patients. In DP settings, it also facilitated a more individualised approach that was less bound to a specific therapeutic environment (e.g., better embedding treatment in patients’ home environment/lives, adjusting treatment expectations/boundaries). Additionally, DP and IP clinicians described innovation and creativity; many wished for the newly created groups to continue. Taken together, these findings suggest the pandemic instigated a necessary re-consideration of treatment-as-usual [21] and afforded greater accessibility in ways that might have otherwise not been tried. Given the increasing demand on ED services and emphasis on individualised support, improving accessibility and efficacy may be of merit [16, 22, 41].

### Strengths and limitations

Interview questions relating to COVID-19 were incorporated into an existing topic guide and permitted the opportunity to explore the impact of COVID-19 on clinicians working in specialist ED intensive treatment settings as the pandemic unfolded. Efforts were made to ensure the credibility, dependability, and transferability of the study, through ongoing researcher reflexivity, triangulation and checking; the description of a detailed methodology, and themes supported with ample quotes and descriptions [42]. Clinicians from a range of professional backgrounds with varying lengths of experience in EDs participated, allowing for an array of perspectives to be considered. Although clinicians were all based in the UK, the results of this study may be applicable elsewhere, as the COVID-19 pandemic has impacted ED services across the globe and many have had to adopt similar ways of working (e.g., partial closures, virtual treatment delivery) [5, 8, 43, 44]. All DP services adapted, rather than closed, which is not representative of the wider UK picture, although positively, it highlights the possibility of virtual intensive ED treatment.

## Conclusions

Given the anticipated short- and long-term impact of COVID-19, particularly within already resource-limited contexts, it is helpful to consider the challenges and opportunities that the pandemic created in specialist ED intensive treatment settings. IP and DP services had to adapt treatment-as-usual. In IP services, there was a clear desire to return to ‘normal’, except for continuing (some) virtual carer involvement and support and newly developed groups beyond the pandemic. In DP services, there was also a strong wish to return to ‘normal’, although clinicians appeared more receptive to some virtual intensive DP treatment. This was due to the potential for increased accessibility and more individualised support, which may help reduce the postcode lottery widely associated with ED services [45]. Nonetheless, COVID-19 posed challenges to the continuation of a multidisciplinary approach. Medical, psychological, practical, and nutritional support, as well as carer involvement and fostering social connections, are valued by clinicians, and should remain at the forefront of intensive treatment for severe AN. Further research is required to explore effective and acceptable implementation of therapeutic meal support and practical groups within restricted, and virtual, intensive settings. Overall, this study highlights the challenge of managing risky patients in resource-limited settings and the uncertainty surrounding which intensive treatment may be best suited to which patient when, particularly within the context of virtual DP support.

## Supplementary Information

**Additional file 1**. Topic Guide.

## Data Availability

The datasets generated and/or analysed during the current study are not publicly available but are available from the corresponding author on reasonable request.

## Abbreviations

AN: Anorexia nervosa
BMI: Body mass index
DP: Day patient
ED: Eating disorder
GP: General practitioner
IP: Inpatient
NHS: National Health Service
UK: United Kingdom
